# Interventions to improve medication adherence in adults with mental–physical multimorbidity in primary care: a systematic review

**DOI:** 10.3399/BJGP.2023.0406

**Published:** 2024-06-11

**Authors:** Elena Lammila-Escalera, Geva Greenfield, Ziyang Pan, Dasha Nicholls, Azeem Majeed, Benedict Hayhoe

**Affiliations:** Department of Primary Care and Public Health; Department of Primary Care and Public Health; Department of Primary Care and Public Health; Department of Primary Care and Public Health; Department of Primary Care and Public Health; Department of Primary Care and Public Health, Imperial College London, London.

**Keywords:** medication adherence, multimorbidity, primary care

## Abstract

**Background:**

Medication non-adherence is a notable contributor to healthcare inefficiency, resulting in poor medication management, impaired patient outcomes, and ineffective symptom control.

**Aim:**

To summarise interventions targeting medication adherence for adults with mental–physical multimorbidity in primary healthcare settings.

**Design and setting:**

A systematic review of the literature — published in any language and with any country of origin — was conducted.

**Method:**

MEDLINE, EMBASE, PsycInfo, Web of Science, Cochrane Library, and the Cumulated Index to Nursing and Allied Health Literature — more commonly known as CINAHL — were searched for relevant studies. Data were extracted and synthesised using narrative synthesis. The Effective Practice and Organisation of Care (EPOC) taxonomy was used to classify intervention types. Risk of bias was assessed using the National Heart, Lung, and Blood Institute’s quality assessment tool for controlled intervention studies.

**Results:**

Eleven studies, representing 2279 patients, were included. All interventions examined were classified into one EPOC domain, namely ‘delivery arrangements’. All included studies examined patients who had a physical condition and depression. Seven studies examining interventions focused on coordination of care and management of care processes reported statistically significant improvements in medication adherence that were attributed to the intervention. Four studies considering the use of information and communication technology observed no changes in medication adherence.

**Conclusion:**

Interventions that coordinate and manage healthcare processes may help improve patients’ adherence to medication regimes in those with mental–physical multimorbidity. However, it is still necessary to better understand how digital health technology can support patients in following their medication regimes. As the growing challenges of treating multimorbidity are faced, everyone involved in health services — from providers to policymakers — must be receptive to a more integrated approach to healthcare delivery.

## Introduction

Medication adherence^1^^,^^2^ is *‘the degree to which the person’s behaviour corresponds with the agreed recommendations from a healthcare provider’*.^1^ This description encompasses several behaviours, from seeking medical attention to consuming medication as prescribed.^2^^,^^3^ Non-adherence, therefore, presents health systems with a multifaceted challenge, imposing a significant economic burden globally.^4^ Five interacting dimensions are recognised to affect the ability to adhere to medication, namely:

- social and economic factors;
- healthcare provider-related factors;
- condition-related factors;
- therapy-related factors; and
- patient-related factors.^3^^,^^5^^,^^6^

Socioeconomic status is one of the most frequently explored contributors to poor medication adherence, because of illness severity and primary care accessibility.^2^^,^^7^

Medication non-adherence is particularly challenging for individuals with multimorbidity.^8^^–^10 Multimorbidity is the coexistence of at least two long-term conditions;^11^^,^^12^ it poses unique clinical challenges as patients have coexisting, and potentially interacting, diseases, which may amplify symptoms and discomfort.^13^^,^^14^ Managing multimorbidity is complex and interventions to improve outcomes must be multifaceted.^15^ Consequently, individuals living with multimorbidity often require multiple medications to achieve optimal treatment.^16^^,^^17^ This may result in a considerable burden on these individuals — for example, those with five or more chronic conditions could spend 5–8 hours a day managing these, which could contribute to treatment burden and reduce medication adherence.^18^^,^^19^ Patients may also prioritise certain medications over others, according to disease progression and severity, acceptability or tolerability, and perceived importance.^2^^,^^7^^,^^14^ Ultimately, this can compromise drug safety, and lead to inappropriate prescriptions, adverse drug reactions, and unnecessary medication interactions.^20^^–^22 Additionally, psychiatric treatments have a lower adherence rate than those for physical conditions.^23^ Mental–physical multimorbidity, which includes common mental disorder (CMD), can reduce adherence rates further.^23^^–^26

**Table:** How this fits in

This research builds on existing knowledge by addressing the uncertainty surrounding the effectiveness of interventions targeting medication adherence in adults with mental–physical multimorbidity in primary care. Prior to this review, the landscape lacked a systematic exploration of such interventions for this complex patient population. The findings offer a comprehensive synthesis of the literature, addressing a crucial evidential gap. Clinicians will benefit from a clearer understanding of those interventions that improve medication adherence in adults with mental– physical multimorbidity, which will help to enhance their ability to tailor care to patients and improve patient outcomes.

Previous studies^16^^,^^27^^,^^28^ have proposed many approaches to improving medication non-adherence. However, it is unclear which of these interventions address this issue, and to what extent they do so effectively, to inform best practice and service delivery. To date, no systematic review has assessed the evidence on interventions targeting medication adherence in individuals with mental–physical multimorbidity. This systematic review aimed to answer the following research question: what type of interventions are designed to improve medication adherence for adults with multimorbidity, including CMD, in primary care, and how effective are they?

## Method

This review was conducted in line with recommendations in the *Cochrane Handbook for Systematic Reviews of Interventions*^29^ and reported in accordance with the Preferred Reporting Items for Systematic Reviews and Meta-Analysis (PRISMA) Guidelines.^30^ The protocol for this systematic review was preregistered on PROSPERO (registration number: CRD42022332974).

### Eligibility criteria

The population, intervention, comparison, outcome, and study design — often referred to as PICOS — framework^31^ was used to formulate eligibility criteria. Studies were not excluded based on published language or country of origin. Studies with a quantitative randomised controlled trial design were eligible for inclusion if they considered adult populations with multimorbidity, including at least one chronic condition and at least one CMD comorbidity, presenting to primary care. Results from pilot studies were also eligible. Individuals with major depression — namely, a severe mental illness — were included in this systematic review for comprehensiveness. Studies were required to compare interventions targeting medication adherence in primary care with usual or standard care, or care without therapeutic components, and had to consider patient medication/treatment/therapy adherence rate as a primary or secondary outcome. Measures of effect included:

- self-reported questionnaires or structured interviews;
- therapeutic drug monitoring;
- electronic devices; and
- prescription pick-up/refill rates.

### Search strategy

#### Information sources

Articles were identified through searches of the electronic databases MEDLINE, EMBASE, PsycInfo, Web of Science, Cochrane Library, and Cumulated Index to Nursing and Allied Health Literature — more commonly known as CINAHL — from January 2000 until May 2022. The search strategy was initially constructed for MEDLINE (Supplementary Table S1), but was later appropriately adapted for use with the other databases searched.

#### Selection process

After deduplication, the titles and abstracts of the eligible articles were independently screened against the inclusion and exclusion criteria by two reviewers. Conflicts were resolved by discussion. This process was repeated for full-text examination.

### Data collection process

Data relevant to the study question were independently extracted from each qualified study by one of the reviewers who had conducted the screening and validated by the other. These data were summarised in a tabular format. Data extracted included the intervention under investigation, sociodemographic factors, and intervention effectiveness.

### Study risk of bias assessment

The same two reviewers independently assessed the included studies for risk of bias using the National Heart, Lung, and Blood Institute’s study quality assessment tool for controlled intervention studies.^32^ Studies were not excluded based on quality assessment.

### Synthesis methods

A meta-analysis was considered impractical, because of the anticipated heterogeneity of interventions. Consequently, a narrative synthesis approach was conducted, utilising the Effective Practice and Organisation of Care (EPOC) taxonomy^33^ to classify the interventions reported. There are four categories:

- delivery arrangements;
- financial arrangements;
- governance arrangements; and
- implementation strategies.

## Results

### Study selection

**Figure 1.**
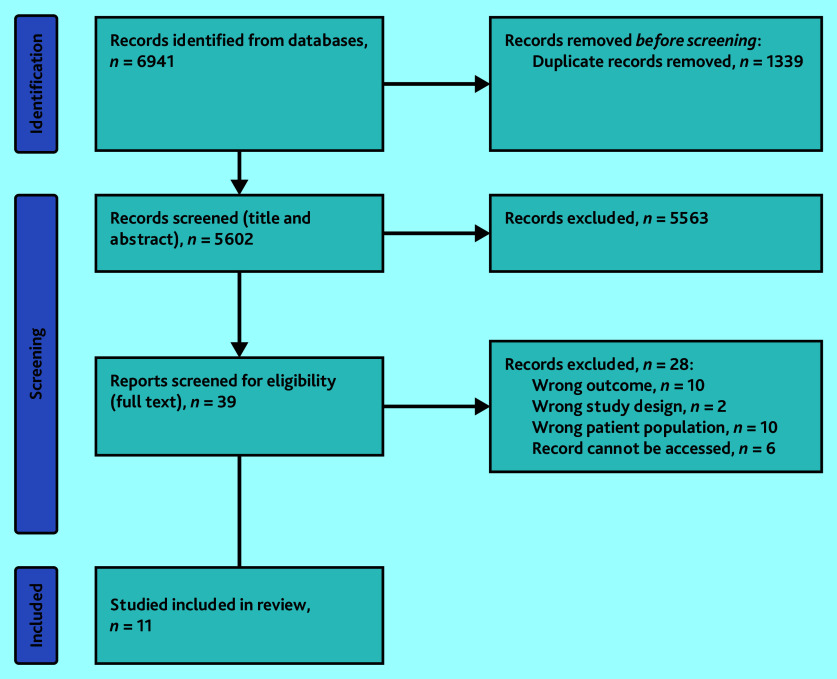
Systematic review flow chart.

Figure 1 outlines the review process. Searches identified 6941 studies; of 39 selected for full-text screening, a total of 28 were excluded because of outcomes, patient population, study design, or that the record could not be accessed. As a result, 11 randomised controlled trials^34^^–^44 were included in this review.

### Study characteristics

Of the 11 trials (Supplementary Table S2), nine^34^^–^36^,^^38^^–^42^,^^44^ were conducted in the US, one^37^ in Australia, and one^43^ in South Africa. Six articles^34^^–^36^,^^38^^,^^43^^,^^44^ targeted medication adherence as a primary outcome. These 11 studies recruited 2279 adults with multimorbidity and CMD. Disease combinations included hypertension, diabetes, HIV/AIDS, and coronary heart disease, all in combination with depression.

Three studies^40^^,^^42^^,^^44^ were deemed as being of good quality, five studies^36^^,^^37^^,^^39^^,^^41^^,^^43^ were considered to be of fair quality, and three studies^34^^,^^35^^,^^38^ were assessed as being of poor quality (Supplementary Table S3). Reasons for the poorer ratings included a failure to meet an adequate randomisation requirement, not specifying the randomisation method, not specifying the treatment allocation concealment, or not reporting the blinding of the researchers.

### Intervention classification

All 11 trials^34^^–^44 assessed interventions that could be classified as delivery arrangement interventions, according to the EPOC taxonomy. Of the interventions reported, the most frequent EPOC subcategories of the delivery arrangement domain that were explored were the:

- coordination of care and management of care processes; and
- use of information and communication technology (ICT).

### Interventions focused on coordination of care and management of care processes

Seven studies^34^^–^36^,^^40^^,^^41^^,^^43^^,^^44^ examined interventions that targeted the coordination of care and the management of care processes. These interventions were multifaceted, with several components spanning EPOC subcategories. These components included:

- case management;
- integration;
- shared care;
- shared decision making; and
- teams.

For instance, three studies^34^^–^36 assessed an integrated depression and chronic-disease treatment intervention by utilising an integrated care manager as a liaison between the physician and patient. The role of this intermediary case management was to work with the patient to address the factors of adherence and effectively communicate the rationale behind antidepressant and antihypertension medication use. Similarly, the multicondition collaborative care management intervention proposed by Lin *et al*^41^ aimed to coordinate care using a care manager.

The nurse-led intervention detailed by Safren *et al*^43^^,^^44^ integrated adherence counselling with traditional cognitive behavioural therapy (CBT) techniques for treating depression and antiretroviral therapy adherence. This programme commenced with problem solving, employing case studies (Life-Steps) to prepare participants for subsequent CBT sessions. Additional resources offered to participants included reminders, graphic aids, and self-care and physical-activity techniques.

Katon *et al*^40^ focused on shared care, decision making, and team collaboration. Nurses collaborated with other health professionals, such as psychiatrists, who would co-review medication use, provide recommendations, and work closely with patients to establish patient-centred goals. A stepped-care algorithm was also employed, whereby participants who did not initially meet goals had to choose their next treatment plan during a second phase of the intervention.

### ICT interventions

Four studies^37^^–^39^,^^42^ utilised ICT and telemedicine to deliver care. Clarke *et al*^37^ assessed the delivery of their self-management CBT programme through a web-based platform. Targeting the improvement of social and occupational functioning, participants were required to complete a minimum set of CBT modules. Moreover, the platform offered self-monitoring reminders, home practice activities, and motivational statements. This intervention was entirely self-guided; the other ICT interventions were guided by health professionals.

Both Himelhoch *et al*^39^ and Piette *et al*^42^ examined the impact of CBT-based interventions delivered via telephone. In contrast, the trial by Cohen *et al*^38^ assessed an intervention comprising electronic-device monitoring and consultation; this pharmacist-led telehealth management programme involved the deployment of an electronic device (Health Buddy^®^) to facilitate communication — it sent individualised daily reminders to patients to take their medications.

### Reported outcomes

All 11 studies collated reported medication adherence as an outcome, with eight^34^^–^39^,^^42^^,^^44^ also reporting mental health outcomes and five^34^^–^37^,^^42^ reporting physical health outcomes (Box 1). Of the seven studies assessing interventions based on coordination of care and management of care processes, six^34^^–^36^,^^40^^,^^43^^,^^44^ reported improved medication adherence, four^34^^–^36^,^^44^ reported improvements in mental health outcomes, and three^34^^–^36 reported improvements in physical health outcomes. Of the four studies examining ICT interventions, two^38^^,^^39^ reported maintained medication adherence, and two^37^^,^^42^ reported improved mental health outcomes only.

**Box 1.** Reported outcomes, by EPOC taxonomy intervention category^33^

**Intervention focus**	**Medication adherence**	**Mental health outcomes**	**Physical health outcomes**
**Coordination of care and management of care processes**	Three studies^34^^–^36 reported that, for their respective outcome assessments, the intervention group exhibited a higher proportion of patients who had ≥80% adherence to medication, compared with their control participants. Three studies^40^^,^^43^^,^^44^ reported that intervention participants had significantly greater medication adherence than the control group. One of these studies^44^ recorded that adherence gains were not maintained at 8 months. A further study^41^ reported that, although there were no differences observed for medication adherence, adjustment rates were higher among the intervention group, relative to the control	Four studies^34^^–^36^,^^44^ reported significant reductions in depression status and depressive symptoms, compared with their control participants	Three studies^34^^–^36 reported better physical chronic-disease indices compared with their control participants
**ICT**	Two studies^38^^,^^39^ observed that medication adherence was maintained in the intervention participants, in comparison with the controls. Two studies^37^^,^^40^ reported no differences in medication adherence between intervention and control participants	Two studies^37^^,^^42^ reported significant reductions in depressive symptoms. In contrast, one study^39^ observed that both intervention and control group participants experienced significant reductions in depressive symptoms. One study^38^ reported that there were no significant changes for both groups in terms of depression scores	One study^37^ observed that intervention participants experienced no differences in blood glucose monitoring. Another study^42^ reported no differences in Hba1c levels between the intervention and control groups

*EPOC = Effective Practice and Organisation of Care. Hba1c = glycated haemoglobin. ICT = information and communication technology.*

## Discussion

### Summary

Current interventions show a potential to improve medication adherence in adults with mental–physical multimorbidity in primary care, despite substantial heterogeneity in participant and intervention characteristics. Trials that assessed interventions focused on the coordination of care and care processes showed improvements across all outcomes, supporting the implementation of this intervention type for this complex patient population. In contrast, studies examining the efficacy of ICT interventions reported conflicting findings.

### Strengths and limitations

To the authors’ knowledge, this is the first systematic review to examine the effectiveness of interventions to improve medication adherence for adults with multimorbidity including CMD. This review addresses a crucial evidential gap for this group, presenting valuable insight to improve service delivery. Moreover, the focus on RCTs ensured that the evidence generated was of high quality, as RCTs are recommended when establishing a causal relationship.^45^

Despite an extensive search, only 11 articles were included because of strict eligibility criteria. Only depression plus a limited number of physical comorbidities were trialled in the studies eligible, restricting applicability across other chronic diseases and mental disorders. Few countries were represented, with all trials conducted in countries with English as their primary language; however, it is important to note that variation in terminology and intervention description, due to differences in language or culture, may have led to some studies being overlooked in the search. This review is also vulnerable to the limitations of the various methods used to quantify adherence, such as self-report measures.^46^

There are recognised limitations in employing the EPOC taxonomy as a guide in synthesis,^33^ and interventions to improve outcomes for individuals with multimorbidity are often multifaceted and have a potential for overlap in categorisation. Furthermore, the interventions demonstrated a broad range of characteristics, varying from marked structural changes in the healthcare team to patient-level amendments; consequently, causality on any outcome cannot be attributed to a specific intervention component. Finally, no studies from the UK were identified. This may have an impact on the contextual adaptability of the interventions proposed, limiting generalisability for widespread implementation in UK settings.

### Comparison with existing literature

This review reaffirms previous conclusions that, despite successes with care coordination and care management processes, evidence for widespread implementation of these interventions remains undeveloped.^47^^–^50 However, unlike previous work,^47^^–^50 this review investigates the efficacy of interventions to improve medication adherence for this unique patient population.

Some of the interventions examined that were reported as successful at improving medication adherence have also been successful when assessed for suitability in other contexts across a variety of outcomes —for example, team care has been used in the treatment and management of other conditions, such as depression and anxiety, fostering improved outcomes in participants, and contributing to the potential usefulness of the intervention throughout the healthcare system.^51^ Previous research^52^^,^^53^ has explored the relationship between care coordination and medication adherence; although the exact mechanism of association is not fully understood, it is likely influenced by fostering a robust relationship between the patient and the professional.^54^^,^^55^ In addition, evidence indicates that an ongoing partnership may improve patient satisfaction, trust, and communication.^56^

### Implications for research and practice

This systematic review identified that the dominant intervention type assessed targeted service delivery arrangements. Trials assessing coordination of care interventions reported improvements in medication adherence as well as mental and physical health outcomes. In contrast, ICT interventions failed, overall, to improve outcomes for trial participants. Additional resources must, consequently, be allocated to foster the creation, testing, and implementation of interventions that aim to integrate care to improve outcomes for this complex group of patients.

To mitigate the impending challenges associated with multimorbidity, health services, providers, and policymakers must be receptive to adopting alternative approaches to care. National guidelines and policies should be reviewed to ensure alignment with best practices for this complex patient population and to support the implementation of novel ideas for change. This review presents several interventions that could be utilised to improve medication adherence, either as a reference for care delivery or as a foundational basis for additional development. Primary care providers should embrace the evidence presented in this review to reinforce care management processes and coordinate efforts to improve outcomes. The patient–provider relationship should also be prioritised when formulating strategies to enhance medication adherence for this complex patient population. The benefit of investment in medication non-adherence strategies could markedly outweigh any associated short-term expenses associated with staffing, cost, and capacity constraints.^4^

Decision makers should also grasp the opportunity to use digital health technologies and integrate them into usual service delivery. Despite conflicting evidence, the MyCompass^37^ platform and the Health Buddy^®^^38^ device represent how digital health technologies may improve outcomes and alleviate associated burdens for the provider. However, comprehensive training, acceptance, communication, and organisational stability must be given special consideration to support the successful implementation of digital health technologies and ensure optimal outcomes.^57^

There is a lack of evidence concerning which existing interventions improve medication adherence for adults with mental–physical multimorbidity in primary care and to what extent they do so. Further research is urgently required to expand the sparse evidence base for interventions supporting the care of this complex group of patients. Most of the studies collated in this review focused solely on a specific combination of chronic disease and CMD; subsequently, more trials should actively include participants with various comorbidity combinations. This would provide greater insight into the viability of widespread implementation, whereby adults with multimorbidity present to primary care with varied combinations of chronic disease. Future research should also conduct sub-group analyses to assess how variables associated with adults with multimorbidity affect the efficacy of these interventions. As an example, only one study in this review recruited African American participants. Special focus should be given to age, ethnicity, and socioeconomic status, as the literature on these factors is limited. By neglecting to incorporate these variables into the analysis, appropriate evidence will not be generated, potentially resulting in inadequate care.

Furthermore, the interventions trialled in this review may not be suitable for implementation across all global contexts. It would be advantageous if trials examined efficacy in other contexts and countries to ensure the interventions’ contextual adaptability. Economic evaluations to determine cost-effectiveness would also be beneficial by providing decision makers with additional operational information for reaching a consensus on feasibility.
